# Deficiency of the p53/p63 target *Perp *alters mammary gland homeostasis and promotes cancer

**DOI:** 10.1186/bcr3171

**Published:** 2012-04-20

**Authors:** Rachel L Dusek, Jamie L Bascom, Hannes Vogel, Sylvain Baron, Alexander D Borowsky, Mina J Bissell, Laura D Attardi

**Affiliations:** 1 Division of Radiation and Cancer Biology, Department of Radiation Oncology, Stanford University School of Medicine, Center for Clinical Sciences Research, Room 1240, 269 Campus Drive, Stanford, CA 94305, USA; 2 Life Sciences Division, Ernest Orlando Lawrence Berkeley National Laboratory, 1 Cyclotron Road, Berkeley, CA 94720, USA; 3 Department of Pathology, Stanford University School of Medicine, R241, 300 Pasteur Drive, Stanford, CA 94305, USA; 4 Center for Comparative Medicine, Department of Medical Pathology, University of California, Davis, County Road 98 and Hutchinson Drive, Davis, CA 95616, USA; 5 Division of Radiation and Cancer Biology, Department of Radiation Oncology and Department of Genetics, Stanford University School of Medicine, Center for Clinical Sciences Research Room 1255, 269 Campus Drive, Stanford, CA 94305, USA

## Abstract

**Introduction:**

*Perp *is a transcriptional target of both p53 during DNA damage-induced apoptosis and p63 during stratified epithelial development. *Perp-/- *mice exhibit postnatal lethality associated with dramatic blistering of the epidermis and oral mucosa, reflecting a critical role in desmosome-mediated intercellular adhesion in keratinocytes. However, the role of Perp in tissue homeostasis in other p63-dependent stratified epithelial tissues is poorly understood. Given that p63 is essential for proper mammary gland development and that cell adhesion is fundamental for ensuring the proper architecture and function of the mammary epithelium, here we investigate Perp function in the mammary gland.

**Methods:**

Immunofluorescence and Western blot analysis were performed to characterize Perp expression and localization in the mouse mammary epithelium throughout development. The consequences of *Perp *deficiency for mammary epithelial development and homeostasis were examined by using *in vivo *mammary transplant assays. Perp protein levels in a variety of human breast cancer cell lines were compared with those in untransformed cells with Western blot analysis. The role of Perp in mouse mammary tumorigenesis was investigated by aging cohorts of *K14-Cre/+;p53^fl/fl ^*mice that were wild-type or deficient for *Perp*. Mammary tumor latency was analyzed, and tumor-free survival was assessed using Kaplan-Meier analysis.

**Results:**

We show that Perp protein is expressed in the mammary epithelium, where it colocalizes with desmosomes. Interestingly, although altering desmosomes through genetic inactivation of *Perp *does not dramatically impair mammary gland ductal development, *Perp *loss affects mammary epithelial homeostasis by causing the accumulation of inflammatory cells around mature mammary epithelium. Moreover, we show reduced Perp expression in many human breast cancer cell lines compared with untransformed cells. Importantly, *Perp *deficiency also promotes the development of mouse mammary cancer.

**Conclusions:**

Together, these observations demonstrate an important role for Perp in normal mammary tissue function and in mammary cancer suppression. In addition, our findings highlight the importance of desmosomes in cancer suppression and suggest the merit of evaluating Perp as a potential prognostic indicator or molecular target in breast cancer therapy.

## Introduction

The vast majority of human cancers arise from epithelial tissues. Understanding cancer development therefore relies on defining the molecules and pathways that support the normal structure and function of epithelia, and how these are disrupted during carcinogenesis. The epithelium of the mammary gland represents a particularly interesting model for epithelial function, as it undergoes recurring dynamic alterations to hew an architecture and physiology specific for different developmental stages. Repeated cycles of mammary epithelial cell proliferation, differentiation, and apoptosis contribute to remodeling of the mammary epithelium, dictating the specific structure and function of the mammary gland throughout morphogenesis. Proper regulation of the processes controlling mammary epithelial form and function is critical, as its dysregulation can impair the normal architecture or behavior of the mammary epithelium, leading to cancer development [1].

Proper adhesion of cells to one another as well as to the substratum is the key for promoting the polarized organization and integrity of the mammary epithelium [2]. Cell- in epithelia is facilitated by two major classes of protein complexes: adherens junctions and desmosomes. Adherens junctions promote the cell-cell adhesion required for organizing epithelial cells into sheets and are fundamental for allowing dynamic rearrangements of epithelia necessary for maintaining homeostasis. In the mammary gland, adherens junctions are important for normal tissue development, function, and organization [3-6]. Desmosomes, which are particularly important for reinforcing intercellular adhesion and enabling tissues to resist mechanical stresses, are crucial for promoting adhesion and epithelial integrity in the epidermis. Desmosomes comprise transmembrane cadherin proteins [desmogleins (Dsg) 1 to 4 and desmocollins (Dsc) 1 to 3] that form an adhesive interface at the cell surface, and proteins such as plakoglobin (Pg) and desmoplakin (Dp), which indirectly connect cadherins to the intermediate filament cytoskeleton [7]. Although desmosomes are present within and between cells in both layers of the mammary epithelium [8,9], the contribution of desmosomes to normal mammary epithelial morphogenesis and function has remained unclear. Cell-culture experiments have suggested a role for desmosomal cadherin-mediated adhesion in some aspects of mammary epithelial morphogenesis and organization [9], but desmosome function in the mammary gland has not been examined *in vivo*.

In addition to regulating normal function of tissues, cell-cell adhesion is well established as a tumor-suppression mechanism. Extensive genetic data from mouse models and human cancers have demonstrated a role for adherens junctions in suppressing cancer progression [10], and reduced expression of adherens junction proteins is often associated with poor clinical outcome for cancer patients [11-15]. In contrast, the role for desmosomes in cancer is less well understood, although more and more evidence supports the idea that desmosomes can inhibit carcinogenesis [16]. In particular, two recent physiologically relevant studies of mice with conditional knockout of desmosomal component-encoding genes indicate a key role for desmosomes in suppressing cancer [17,18].

Perp (p53 apoptosis effector related to PMP-22) is a tetraspan-membrane protein essential for desmosome function in the skin and oral mucosa [19,20]. *Perp *was originally identified as a transcriptional target of the p53 tumor suppressor induced specifically during apoptosis and was subsequently found to be regulated by another member of the p53 family, p63 [20], a master regulator of the development of stratified epithelia, such as epidermis [21-23]. *Perp-*null mice exhibit severe blistering of the skin and oral cavity, which likely contributes to their early postnatal lethality. This blistering phenotype is reminiscent of human diseases or mouse models in which desmosome-mediated intercellular adhesion is compromised [24,25]. Immunogold electron microscopy unequivocally demonstrated the localization of Perp to desmosome junctions, and ultrastructurally abnormal desmosomes were observed in the absence of Perp [20]. Thus, it is clear that Perp plays a crucial role in promoting tissue integrity and homeostasis in the epidermis and oral cavity. This essential role has been underscored by our recent demonstration that conditional deletion of *Perp *in mouse skin promotes UVB-induced skin cancer development and progression [17].

Because the mammary epithelium relies on p63 for proper development [22,23] and because adhesion programs are critical for its morphogenesis [26], we hypothesized that Perp may contribute to mammary gland morphogenesis and function. Here, we tested this hypothesis by characterizing Perp expression in the normal mammary epithelium and examining the consequences of Perp loss in mammary gland development. Because the identified functions of Perp in apoptosis and intercellular adhesion, as well as its identification as a suppressor of skin cancer, suggest a potential role for Perp in breast cancer suppression, we additionally examined this possibility by using a mouse mammary cancer model. Collectively, our findings reveal a role for Perp in mammary gland homeostasis and tumor suppression, thus advancing our understanding of the contribution of desmosomes to these processes.

## Materials and methods

### Cell culture

Primary mammary epithelial cells were isolated from mouse mammary glands essentially as described [27]. In brief, two to four 4^th ^mammary glands were dissected from virgin adult females and minced with razor blades, and then incubated in a warm, sterilized solution of 0.2% trypsin (Gibco/Invitrogen, Carlsbad, CA, USA), 0.2% collagenase A (Roche Applied Science, Indianapolis, IN, USA), 5% fetal bovine serum (FBS; Omega Scientific, Tarzana, CA, USA), and 5 mg/ml gentamycin (Gibco/Invitrogen) in DMEM/F12 media (Gibco/Invitrogen) with shaking at 37°C for 30 minutes. Samples were centrifuged for 10 minutes at 1,500 rpm, and the top layer and the bottom pellet were each resuspended in 10 ml of DMEM/F12 and centrifuged again. The resulting pellets were combined in 4 ml media with 40 μl of 2 U/ml Dnase (Invitrogen) and agitated for 2 to 5 minutes at room temperature. Media was added to 10 ml, and the sample was centrifuged for 10 minutes at 1,500 rpm. The pellet was retained and resuspended in another 10 ml of DMEM/F12. The sample was pulsed at 1,500 rpm, and the pellet was retained. This was repeated 6 times, and the pellet was then resuspended in Growth Medium (0.1% ITS (1000×) (Sigma Chemical Corp., St. Louis, MO, USA), 0.005% 100 μg/ml EGF (Sigma Chemical Corp.), 5% FBS, 0.1% 50 mg/mL gentamycin, 1% penicillin/streptomycin (Gibco), in DMEM/F12), and plated at the desired concentration into 60-mm tissue-culture plates or onto coverslips. Medium was changed the next day and every second day thereafter.

The mouse mammary epithelial cell line, EpH4, was cultured in DMEM/F12 supplemented with 5 μg/ml insulin (Sigma Chemical Corp.) and 50 μg/ml gentamycin. Primary mouse keratinocytes were isolated from newborn pups and cultured as described [28]. Primary human keratinocytes were isolated and cultured as described [28]. The following cell lines were obtained from James Ford, Stanford University, and cultured in the corresponding media: MCF7 (DMEM (Gibco) + 10% FBS), MCF10A (equal parts DMEM + glutamine and Ham F-12 (Gibco) + 5% horse serum (Lonza), 1% penicillin/streptomycin, 20 ng/ml EGF, 10 μg/ml insulin (Sigma Chemical Corp.), and 0.5 μg/ml hydrocortisone (Sigma Chemical Corp.)), SUM149PT (Ham's F-12 with 10% FBS, 5 μg/ml insulin and 1 μg/ml hydrocortisone), HCC38, HCC1937, HCC1806, BT549, and T47D (RMPI1640 (Gibco) + 10% FBS).

### Antibodies, immunofluorescence, immunohistochemistry, and Western blotting

Immunofluorescence was performed as described on sections of mouse tissues fixed in 4% paraformaldehyde for 2 hours at room temperature or in 10% formalin overnight at room temperature, and then embedded in paraffin and processed for histologic analysis [17]. Alternately, immunofluorescence was performed on primary mammary epithelial cells isolated from mouse mammary gland, as described earlier, and fixed onto coverslips with ice cold methanol for 5 minutes. Before blocking, samples to be stained for Perp were incubated in a 10 mg/ml solution of proteinase K (Sigma Chemical Corp.) for 10 minutes at room temperature, and then washed. To demonstrate Perp antibody specificity, Perp antibody was diluted 1:50 in PBS containing 2 μg/μl Perp C-terminal crude peptide (containing the last 20 amino acids of the Perp protein sequence [PNYEDDLLGAAKPRYFYPPA]). This was mixed for 2 hours at room temperature, and then applied to samples for immunofluorescence analysis. Immunohistochemistry was performed on mammary gland whole mounts, which were paraffin embedded and sectioned, according to standard protocols.

Slides were analyzed on a Leica DM6000B fluorescence microscope (Leica Microsystems, Buffalo Grove, IL, USA), and image acquisition was performed using a Retiga Exi camera (Qimaging, Surrey, BC, Canada) and Image Pro 6.2 software (Media Cybernetics, Bethesda, MD, USA). Primary antibodies used for immunofluorescence/immunohistochemistry include the following: rabbit anti-Perp [20] (1:200), mouse anti-smooth muscle actin (Sigma Chemical Corp.; 1:200; used as a marker for mammary myoepithelial cells [29]), mouse anti-keratin 8 (University of Iowa Developmental Studies Hybridoma Bank, Iowa City, IA, USA; 1:250; used as a marker for mammary luminal epithelial cells [30], mouse anti-desmoplakin clone 115F (gift from David Garrod, University of Manchester; 1:50), chicken anti-plakoglobin 1408 (gift from Kathleen Green, Northwestern University; 1:100), rat anti-CD45 (BD Biosciences, San Diego, CA, USA; 1:800). Secondary antibodies used included FITC goat anti-rabbit (Vector Laboratories, Burlingame, CA, USA; 1:300), Alexa 546 donkey anti-mouse (Invitrogen; 1:300), and TRITC goat anti-chicken (Vector Laboratories; 1:300).

Whole-cell lysates were prepared as described [20]. Equal amounts of total protein for each sample were subjected to Western blot analysis using standard protocols. Primary antibodies used for immunoblotting are as follows: rabbit anti-Perp [20] (1:200), mouse anti-desmoplakin clone 115F (1:500), mouse anti-desmoglein 1/2 clone 4B2 (1:1,000), chicken anti-plakoglobin 1408 (1:5,000), mouse anti-desmocollin 2 (Abcam, Cambridge, MA, USA; 1:5,000), mouse anti-β actin peroxidase conjugate (Sigma Chemical Corp.; 1:1,000), mouse anti-GAPDH (Fitzgerald Laboratories, Acton, MA, USA; 1:15,000). Secondary antibodies included HRP goat anti-mouse (1:5,000), HRP goat anti-chicken (1:5,000), HRP goat anti-rabbit (1:5,000; Jackson Immunoresearch, West Grove, PA, USA).

### Mammary gland whole mount

The 4^th ^mammary glands were dissected from female mice at the appropriate developmental stage and fixed overnight in Carnoy's solution (3 parts 100% ethanol to 1 part glacial acetic acid). The fixative was discarded, and the tissue was incubated in 70% ethanol for 15 minutes, washed in running tap water for 5 minutes, followed by an overnight incubation at room temperature in carmine alum stain (0.2% carmine aluminum lake (VWR, Radnor, PA, USA), 0.5% aluminum potassium sulfate (VWR)). The tissue was incubated successively in 70% ethanol for 15 minutes, in 90% ethanol for 15 minutes, and in 100% ethanol for 15 minutes, and then immersed in xylene overnight. Coverslips were mounted on the slides with Permount (Fisher Scientific, Pittsburgh, PA, USA) and imaged on a Leica SD6 dissecting microscope using a Leica DFC280 digital camera (Leica Microsystems) and Photoshop software.

### Histology

The 5-μm sections of formalin-fixed, paraffin-embedded mammary glands and tumors were stained with hematoxylin and eosin. Slides were examined with bright-field microscopy using the microscope and imaging software described for immunofluorescence and immunohistochemistry analysis.

### Mammary transplants

This procedure was performed essentially as described [31]. Recipient female mice, 3 weeks of age, were anesthetized with subcutaneous injection of 0.5 mg avertin (Sigma Chemical Corp.)/gram mouse weight, and 2.5 mg/kg carprofen (Pfizer, New York, NY, USA) as an analgesic. The 4^th ^mammary glands were cleared, and the donor tissue (rudimentary mammary epithelium dissected from the 4^th ^mammary glands of newborn female mice) was inserted into a pocket created in the cleared mammary fat pad. Incisions were closed with wound clips. Mice were returned to a clean and warmed cage and observed until recovered from the anesthesia. Transplanted mammary glands were examined with whole-mount histology at different stages of development. Alternately, MECs were isolated from mature mammary glands transplanted with wild-type or *Perp-/- *newborn mammary epithelium, as described earlier.

### Tumor study

*K14-Cre/+ *mice [32] (C57BL/6 and 129 mixed background; gift of Steven Artandi, Stanford University) were bred to *Perp^fl/fl ^*and *p53^fl/fl ^*mice (FVB background; Stock B6.129P2-*Trp53^tm1Brn^*/J, Jackson Laboratory, Bar Harbor, ME, USA) to generate cohorts of *K14-Cre/+;p53^fl/fl^;Perp^fl/+^*, and *K14Cre/+;p53^fl/fl ^*littermates. Sixteen mice of each group were monitored for tumor incidence by palpation and visual inspection and sacrificed when tumors reached 1.5 cm in size or when moribund. Tumor-free survival curves were generated by Kaplan-Meier analysis, and the Log-rank test was performed to determine significance. Tumor latency times were recorded for all palpated tumors, and all palpated mammary tumors, for mice of each cohort, and analyzed with an unpaired two-tailed *t *test to determine significance.

### Ethics statement

The use of murine subjects in this research has been approved by the Institutional Animal Care and Use Committee/Administrative Panel on Laboratory Animal Care at Stanford University (Protocol number 10382). The Institutional Review Board of the Stanford Human Research Protection Program reviewed the use of human subjects in this study and determined the research to be exempt.

## Results

### Perp is expressed in the mammary epithelium

**Figure 1:**
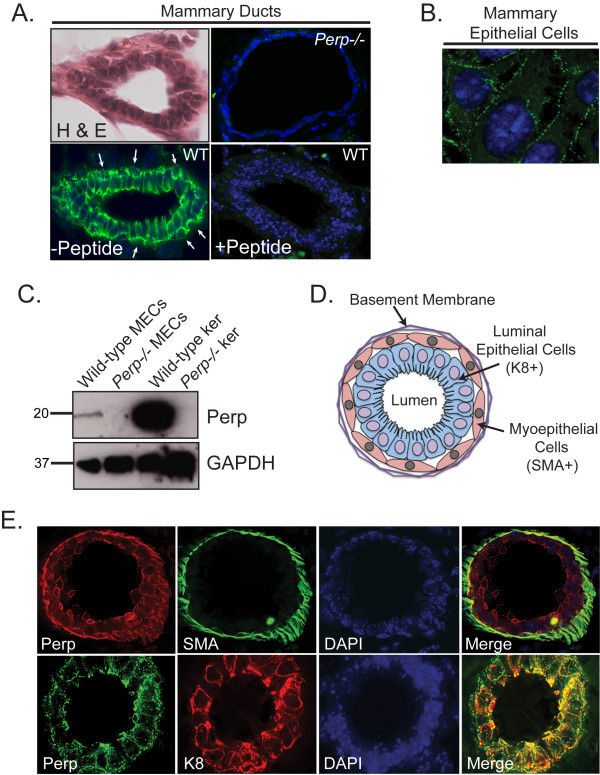
**Perp is expressed in the mammary epithelium**. **(A) **Upper left, Representative hematoxylin and eosin (H&E)-stained cross section of a wild-type virgin mouse mammary duct to demonstrate morphology. Lower left, Perp immunofluorescence of wild-type virgin mouse mammary gland sections, preincubated without (-Peptide) or Lower right, with the peptide (+Peptide) used to generate the Perp antibody, to demonstrate specificity of antibody staining. Arrows indicate potential Perp staining in myoepithelial cells. Upper right, A *Perp-/- *mammary gland section stained for Perp is also shown as a control. **(B) **Perp immunofluorescence image of stained cultured wild-type primary mammary epithelial cells. **(C) **Western blot analysis of wild-type and *Perp-*null mammary epithelial cells (MECs) and keratinocytes (ker) showing levels of Perp. GAPDH serves as a loading control. **(D) **Schematic diagram of a mammary duct in cross section, adapted from Adriance *et al. *[8]. **(E) **Dual-labeled immunofluorescence of wild-type virgin mouse mammary gland sections detailing localization of Perp and markers of the myoepithelium and luminal epithelium, smooth muscle actin (SMA), and keratin 8 (K8), respectively. Nuclei are marked with DAPI staining, and composite images are shown in merged panels. Images are taken at ×630 magnification.

To decipher the function of Perp and desmosomes in the integrity and function of the mammary epithelium, we first examined the localization of Perp in wild-type 14-week-old virgin female mouse mammary glands. With immunofluorescence microscopy, we observed robust Perp staining in the mammary epithelium, with specific localization to puncta at the plasma membrane (Figure 1A). The staining pattern suggests that Perp-positive cells can be found in both layers of the mammary epithelium. The staining is specific for Perp because it can be blocked with a Perp peptide and is absent in *Perp-/- *mammary gland. Immunofluorescence analysis of cultured primary mammary epithelial cells (MECs) further highlighted the punctate Perp staining at cell-cell borders (Figure 1B), a pattern typical of desmosome components. These findings were confirmed with Western blot analysis, in which we clearly detected Perp in MECs isolated from wild-type, but not *Perp-/- *mammary epithelium from adult virgin female mammary glands (Figure 1C). Collectively, these findings provide the first demonstration that Perp protein is indeed expressed in the mammary epithelium.

The ductal mammary epithelium comprises two layers: the luminal epithelial layer facing the lumen of ducts and lobules, and the myoepithelial cell layer surrounding the luminal cells on their basal side and interacting with the basement membrane on the outside edge (Figure 1D) [8]. We were able to distinguish clearly the luminal cell layer from the myoepithelial layer by co-staining for markers of each layer, keratin 8 and smooth muscle actin (SMA), respectively. To define the expression pattern of Perp within these layers, we co-stained mammary gland sections for Perp and SMA to highlight myoepithelial cells [29], and for Perp together with keratin 8 to mark luminal epithelial cells [30] (Figure 1E). We found Perp staining in cells that appeared positive for SMA, as well as in cells that stained positively for keratin 8. These results demonstrate that Perp is expressed in luminal cells of the mammary epithelium and also suggest that Perp is expressed in myoepithelial cells or at the luminal-myoepithelial cell interface.

### Perp is associated with desmosomes in the mammary epithelium

**Figure 2:**
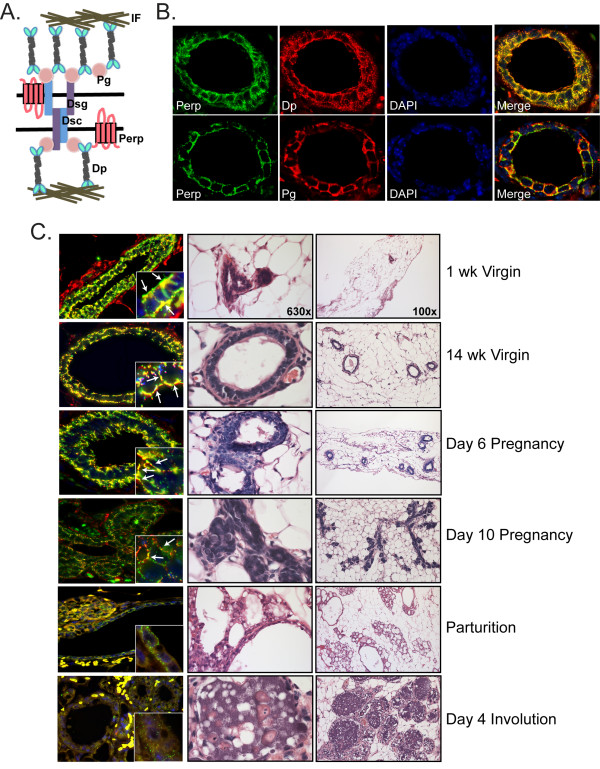
**Perp colocalizes with desmosome proteins**. **(A) **Schematic diagram of the desmosome complex, highlighting several of the major protein components: Dsg, desmoglein; Dsc, desmocollin; Pg, plakoglobin; Dp, desmoplakin; IF, intermediate filaments. The depiction here of Perp's position within the desmosome is speculative, as its direct interacting partners have yet to be defined. **(B) **Dual-label immunofluorescence of wild-type virgin mouse mammary gland sections stained for Perp and the desmosome components Dp or Pg. Nuclei are marked with DAPI staining, and composite images are shown in merged panels. **(C) **Left, Composite images of Perp (green) and Dp (red) immunofluorescence on cross sections of wild-type mouse mammary glands at the indicated stages of development. Insets are shown at high magnification. Arrows indicate areas where Perp and Dp colocalize. Center and right, Representative H&E-stained sections of wild-type mouse mammary glands at the same stages. Images are taken at ×100 and ×630 magnification, respectively.

We previously identified Perp as a novel component of desmosomal cell-cell adhesive junctions in keratinocytes of the epidermis and oral mucosa [20]. The pattern of punctate Perp staining at cell-cell borders that we observed in the mammary epithelial cells (Figure 1A and 1B) is reminiscent of that which we previously reported in keratinocytes, suggesting the possibility that Perp may also play an adhesive role in the mammary epithelium. Indeed, desmosomes (Figure 2A) are found in the mammary epithelium between cells within each layer, as well as between cells of different layers ([8] and our unpublished observations). We examined colocalization of Perp with known desmosome components in adult virgin female mouse mammary glands and observed significant colocalization of Perp with both desmoplakin (Dp) and plakoglobin (Pg), two key desmosomal components (Figure 2B), suggesting that Perp is indeed associated with desmosomes in the mammary epithelium and likely participates in cell-cell adhesion, as it does in the skin.

The mammary epithelium is a dynamic structure that dramatically changes its form and function throughout development [9,33]. To define the involvement of Perp here, we analyzed Perp expression and localization throughout the different stages of mammary gland morphogenesis. We observed that Perp staining generally mirrored that of the desmosome protein, Dp, at all stages examined (Figure 2C). Both proteins were robustly expressed at the plasma membrane of mammary epithelial cells in wild-type virgin mice and during the early stages of pregnancy. As pregnancy proceeded, the levels of both desmosome proteins decreased dramatically, and by parturition, punctate desmosome staining was quite reduced and much less organized. During involution, staining for Perp and Dp continued to be scant. The changing expression of Perp throughout this process is consistent with the previously reported extensive tissue remodeling and dynamic regulation of desmosome proteins that occurs during mammary gland development [34]. Abundant desmosomes are likely needed to anchor cells together and to maintain epithelial integrity and polarity in virgin mammary glands and at the beginning of pregnancy. However, reducing or eliminating adhesive junctions in epithelia of secretory alveoli and small ducts may facilitate the morphologic changes needed for myoepithelial cell contraction and movement of milk through the ductal system during lactation [34].

### *Perp *deficiency perturbs mammary epithelial homeostasis

**Figure 3:**
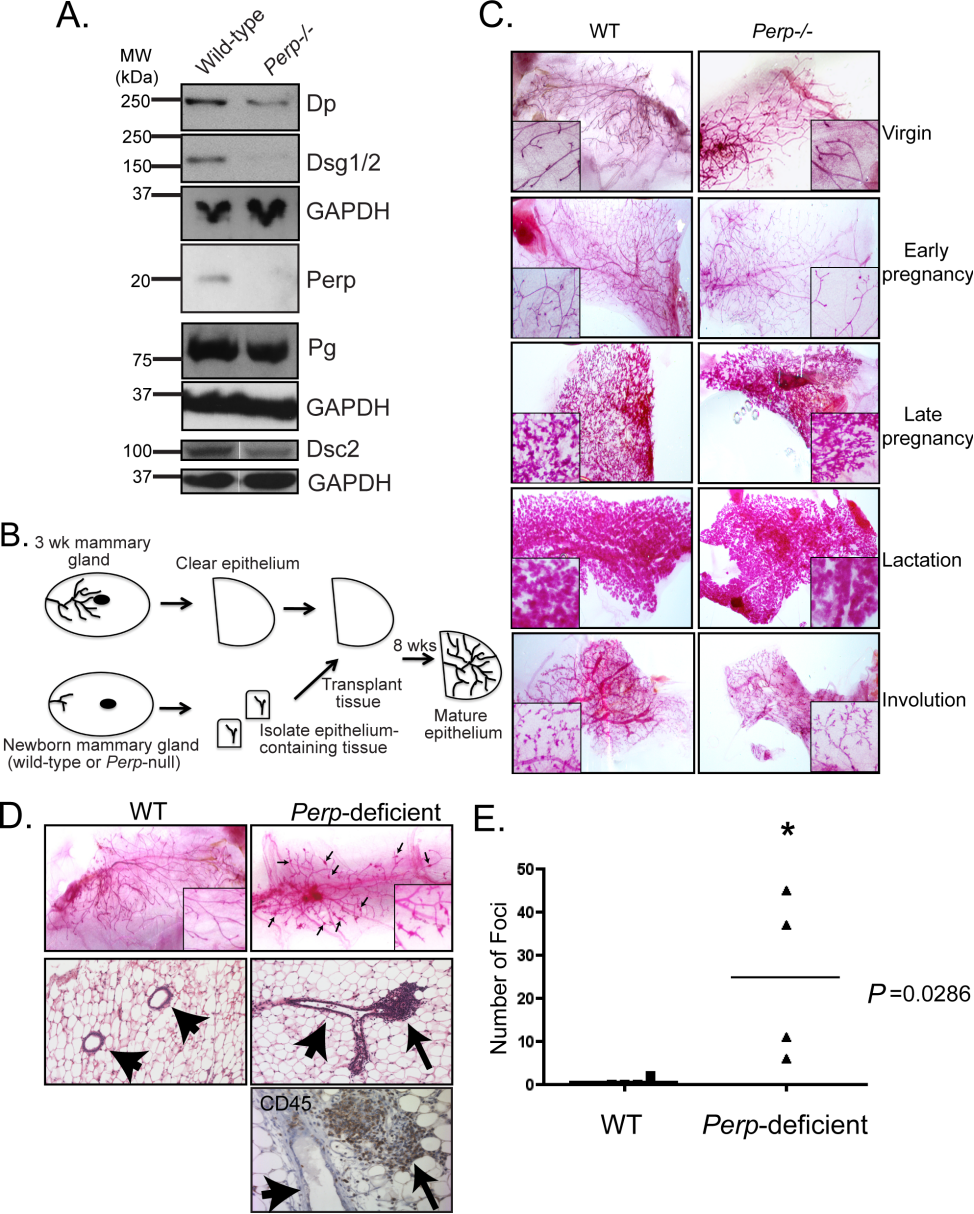
***Perp *deficiency perturbs mammary epithelial homeostasis**. **(A**) Western blot analysis of a variety of desmosomal proteins in wild-type and *Perp-*null mammary epithelial cells. GAPDH serves as a loading control. Dsg, desmoglein; Dp, desmoplakin; Pg, plakoglobin, Dsc, desmocollin. **(B) **Schematic diagram detailing the process of mammary transplantation, adapted from Edwards *et al. *[51]. **(C) **Carmine alum-stained whole-mount preparations of mammary epithelia at different stages. Cleared mammary fat pads were transplanted with wild-type or *Perp*-null mammary epithelium and allowed to develop to the indicated stages: Virgin, 8 weeks old; early pregnancy, day 3.5 after coitus; late pregnancy, day 14.5 after coitus; lactation, day 2 after parturition; involution, day 5 after weaning. **(D) **Whole-mount preparations (top row), hematoxylin and eosin (H&E)-stained sections (middle row), and CD45-stained section (bottom row) of 8-week-old virgin mouse mammary glands transplanted with wild-type or *Perp*-deficient mammary epithelium, showing lymphocytic aggregates (arrows) and mammary ducts (arrowheads). H&E and CD45-stained images were photographed at 100× and 400× magnification, respectively. **(E) **The difference between the numbers of foci in wild-type and *Perp*-deficient mammary whole mounts is graphically depicted. Statistical analysis was performed by using the Mann-Whitney test, for which * indicates statistical significance, *P *= 0.0286, *n *= 4 for each group.

Perp deficiency in the oral mucosa and epidermis causes desmosomal adhesion defects that result in compromised tissue integrity and postnatal death [20]. To determine the consequences of Perp deficiency for desmosomes in the mammary epithelium, we compared the expression of desmosome proteins in whole-cell lysates of wild-type and *Perp-/- *mammary epithelial cells, isolated from transplanted mammary glands. Whereas Perp deficiency did not alter the ability of desmosome proteins to localize properly to the plasma membrane (data not shown), as we previously noted in the skin [20], we observed a reduction in the total levels of Dp, Dsg1/2, and Dsc2 in the *Perp-/- *MECs (Figure 3A), whereas Pg levels seemed less affected by Perp loss. These findings suggest that Perp loss compromises desmosome numbers or integrity in the mammary epithelium.

Given the previously reported experiments showing that desmosome-mediated adhesion contributes to proper mammary epithelial morphogenesis and organization in culture [9], we examined whether the reduction in desmosomal constituents we observed in *Perp-/- *mammary epithelial cells would translate to defects in mammary gland development. Because most *Perp-/- *mice die just after birth [20], we were unable to examine adequately the mammary glands of female *Perp-/- *mice at different developmental stages. Instead, we used an *in vivo *transplantation approach to investigate the importance of Perp and desmosomes for mammary gland development. Mammary transplant assays were performed with mammary epithelial tissue derived from wild-type and *Perp-*/- newborn mice and implanted into the cleared mammary fat pads of syngeneic females (Figure 3B). Whole-mount analysis of mammary epithelia was then performed on the mammary glands of the transplant recipients at specific time points. Despite decreases in desmosome components in *Perp-/- *mammary epithelial cells, we found that *Perp-/- *transplanted epithelium underwent grossly normal morphogenesis through distinct developmental stages, similar to wild-type transplants (Figure 3C). For example, morphometric analysis of the longest duct in each wild-type and *Perp-/- *transplanted mammary gland did not demonstrate a significant difference in duct length or number of side branches between the genotypes (data not shown). Interestingly, however, on close investigation of virgin 8-week-old mice with *Perp-*deficient transplants, we discovered a significantly increased number of abnormal nodules or foci, especially at the bifurcation points of branching ducts, compared with wild-type counterparts (Figure 3D, E). Histologic analysis of the whole-mount samples identified the foci as lymphocytic aggregates, surrounding or sometimes invading the ductal epithelium and associated blood vessels (Figure 3D). Immunohistochemistry for CD45, a leukocyte marker, confirmed the identity of these cells as immune cells. These findings suggest that *Perp *deficiency is associated with enhanced inflammation around the mammary epithelium in the context of this transplant assay, reminiscent of the increase in inflammatory cells we observed in *Perp*-deficient skin after prolonged UVB exposure [17].

### Perp deficiency promotes mammary cancer development

**Figure 4:**
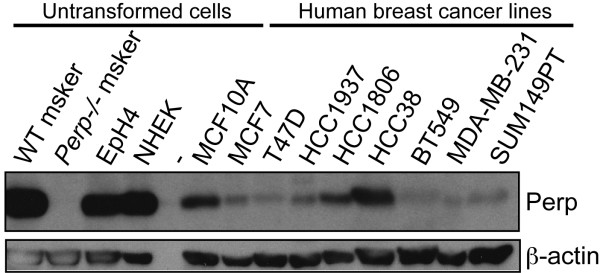
**Many human breast cancer cell lines exhibit reduced Perp expression compared with untransformed cells**. Western blot analysis of Perp levels in multiple human breast cancer cell lines and in untransformed human and mouse mammary epithelial cell lines (MCF10A and EpH4, respectively). Wild-type and *Perp-/- *mouse (msker) keratinocytes and normal human epidermal keratinocytes (NHEK) demonstrated that the antibody recognizes Perp. β-Actin served as a loading control.

The disrupted mammary epithelial homeostasis and enhanced recruitment of inflammatory cells in *Perp*-deficient epithelia we observed here and previously [17] represent intriguing phenotypes because chronic inflammation is known to contribute to cancer development and progression [35]. Given Perp's reported functions in promoting apoptosis and mediating cell-cell adhesion [17,19,20,28], both of which are important tumor-suppression mechanisms, as well as our recent demonstration of Perp in suppressing skin cancer [17], we investigated whether Perp might also suppress breast cancer development. Initially to explore this idea, we examined whether Perp levels decreased during cancer development. Indeed, on examining Perp expression in normal mammary cells and breast cancer cell lines, we found that Perp levels were reduced in the majority of the human breast cancer cell lines tested, relative to the robust Perp levels observed in untransformed human and mouse breast epithelial cell lines (MCF10A and EpH4, respectively) [36] and primary mouse mammary epithelial cells (data not shown; Figure 4). These data demonstrate that Perp levels are diminished in many human breast cancer cell lines, supporting the notion that its loss could contribute to mammary tumorigenesis.

**Figure 5:**
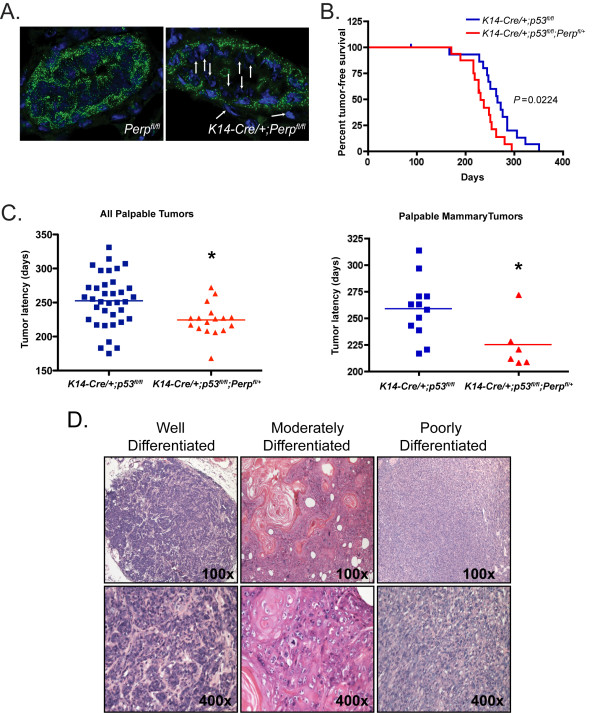
***Perp *deficiency promotes mammary tumorigenesis**. **(A**) Perp immunofluorescence on sections of mammary ducts from *K14-Cre/+;Perp^fl/fl ^*and control *Perp^fl/fl ^*female virgin mice. DAPI marks the nuclei. Arrows indicate cells exhibiting Perp loss. **(B) **Kaplan-Meier analysis graphing overall tumor-free survival, based on assessing all tumor types (breast, skin, and salivary gland tumors), in control *K14-Cre/+;p53^fl/fl ^*(*n *= 16; median survival, 265 days) and *K14-Cre/+;p53^fl/fl^;Perp^fl/+ ^*(*n *= 16; median latency, 230 days) mice. *P *= 0.0224 by the Log-rank test. **(C) **Left, Graph depicting latency of all tumors identified in *K14-Cre/+;p53^fl/fl ^*(*n = *36; mean tumor latency, 257.6 days) and *K14-Cre/+;p53^fl/fl^;Perp^fl/+ ^*(*n *= 17; mean tumor latency, 224.4 days) mice by palpation. Significance (*) was determined by using an unpaired two-tailed *t *test, *P *= 0.0075. Right, Latency for mammary tumors detected by palpation is depicted for *K14-Cre/+;p53^fl/fl ^*(*n *= 12; mean tumor latency, 259 days) and *K14-Cre/+;p53^fl/fl^;Perp^fl/+^*(*n *= 6; mean tumor latency, 225 days) mice. Significance (*) was determined by using an unpaired two-tailed *t *test, *P *= 0.0228. **(D) **Representative H&E images demonstrating the range of differentiation states of mammary tumors observed in *K14-Cre/+;p53^fl/fl ^and K14-Cre/+;p53^fl/fl^;Perp^fl/+ ^*mice. Images were taken at ×100 and ×400 magnification.

To test directly whether *Perp *deficiency promotes mammary cancer development *in vivo*, we used a mouse mammary cancer model, in which the tumor suppressor *p53 *is conditionally inactivated in the mammary epithelium through use of a keratin 14 (K14)-Cre transgene. Such *K14-Cre/+;p53^fl/fl ^*mice develop generally noninvasive mammary carcinomas and carcinosarcomas, as well as squamous cell carcinomas of the skin [37]. This model was previously used to model human invasive lobular breast carcinoma through conditional knockout of the adherens junction protein E-cadherin (*K14-Cre/+;p53^fl/fl^;E-cadherin^fl/fl^)*, which enhanced tumor development and promoted metastasis relative to *K14-Cre/+;p53^fl/fl ^*controls [37]. Because homozygous *Perp *inactivation often causes lethality, we generated conditional *Perp *heterozygotes and took advantage of the *K14-Cre *strain to create *K14-Cre/+;p53^fl/fl^;Perp^+/+ ^*and *K14-Cre/+;p53^fl/fl^;Perp^fl/+ ^*mouse cohorts, in which we could study *Perp*-deficient mammary epithelium. We first queried the efficiency of *K14-Cre *in deleting *Perp *in the mammary epithelium by analyzing Perp expression in adult virgin female *K14-Cre/+;Perp^fl/fl ^*or control mammary glands. Instead of widespread loss of Perp throughout the entire mammary epithelium, *K14-Cre *expression resulted in *Perp *deletion in only a subset of individual cells in a rather stochastic fashion (Figure 5A), as reported [37,38] and recapitulating the way in which gene mutations typically occur in cancer [39]. The control and experimental cohorts from the tumor study were aged for up to 1 year and monitored for tumor development. Mammary glands and skin were palpated twice a week to determine when tumors developed, and the animals were sacrificed when tumors reached 1.5 cm in size or when moribund. We observed a significant reduction in tumor-free survival for *K14-Cre/+;p53^fl/fl^;Perp^fl/+^*mice (median tumor-free survival, 230 days) compared with *K14-Cre/+;p53^fl/fl ^*control mice (median tumor-free survival, 265 days; *P *= 0.0224; Figure 5B). Furthermore, we compared the average latency of tumor initiation in each cohort and found that tumors in *K14-Cre/+;p53^fl/fl^;Perp^fl/+^*mice developed significantly earlier than those in *K14-Cre/+;p53^fl/fl ^*mice (*P *= 0.0075; Figure 5C). This significant finding was also evident when mammary tumors were analyzed separately (*P *= 0.0228; Figure 5C). Histologic characterization of tumors verified that mice of each cohort developed mammary tumors of grades ranging from *in situ *lesions to invasive cancers that were well, moderately, or poorly differentiated. (Figure 5D). Thus, our findings collectively indicate that *Perp *deficiency promotes mammary tumorigenesis. These data further highlight a novel role for Perp and desmosomes as inhibitors of mammary cancer driven by p53 tumor suppressor loss.

## Discussion

We previously identified Perp as a crucial component of desmosomes in the oral mucosa and epidermis, where it acts to promote tissue integrity [20], and as a mediator of p53-dependent apoptosis in response to genotoxic stress [17]. To gain a greater understanding of the function of Perp in tissue homeostasis in other contexts, we investigated a role for Perp in the mammary epithelium, another p63-dependent tissue in which cell adhesion and apoptosis are key for development and homeostasis. Here, we provided the first demonstration that Perp protein is expressed in the mammary epithelium, in a pattern suggesting an adhesive role for Perp in the mammary epithelium, as in the oral epithelium and skin. In addition, our results demonstrate that *Perp *deficiency both perturbs mammary epithelial homeostasis and promotes mammary cancer.

In the mammary epithelium, Perp exhibits a punctate pattern of expression at the plasma membrane and colocalizes with other desmosome components. Our results suggest that Perp is expressed in both layers of the mammary epithelium, in luminal and myoepithelial cells. Perp may be an important factor in regulating desmosome assembly and/or stability in this context, as we found that Perp loss in mammary epithelial cells decreased levels of the desmosome proteins Dp, Dsg, and Dsc, compared with those in wild-type cells. These findings suggest that desmosome function in the mammary epithelium is likely compromised in the absence of *Perp*.

Akin to global Perp deficiency causing impaired desmosome function in the oral mucosa and epidermis, associated with blistering and early lethality [20], Perp loss in the mammary epithelium also compromises mammary gland homeostasis *in vivo *by promoting immune cell recruitment. We demonstrated further that *Perp*-null mammary epithelium is able to realize all stages of mammary gland development without the presence of gross structural abnormalities, suggesting that *in vivo *mammary gland morphogenesis *per se *is not dependent on normal desmosome function. This finding contrasts with a previous study reporting that impairing desmosome-mediated adhesion by simultaneously interfering with the function of both types of desmosomal cadherins, Dsg and Dsc, inhibited mammary epithelial morphogenesis in culture, as seen by the impaired development of alveolar spheres and the inhibition of positional sorting of luminal and myoepithelial cells in aggregates [9]. This observation could suggest that inactivation of two desmosomal cadherins may be more severe than the loss of Perp in the context of mammary epithelial development. Differences between our findings and those of this report may also be due to the experimental design used in each study: we examined primary mouse mammary epithelium *in vivo*, whereas Runswick *et al. *[9] used a mouse mammary cell line in cell-culture experiments.

Because our mammary-transplant assays were performed *in vivo*, we were able to examine the mammary epithelium in its native context. Strikingly, in the context of mammary transplantation, we observed abundant immune cell accumulation around the *Perp*-deficient mammary epithelium in 8-week-old virgin females, but not that of wild-type counterparts. This observation is reminiscent of our previous findings that ablation of *Perp *in the epidermis induced an inflammatory gene signature and that *Perp *deficiency in combination with UV irradiation caused the accumulation of immune cells in the skin [17]. Inflammatory cells are well established to promote tumor progression by producing such factors as cytokines, chemokines, and matrix metalloproteases that can enhance the malignant characteristics of neoplastic cells as well as remodeling the tumor microenvironment to support tumor growth and progression. Indeed, leukocyte accumulation increases during tumor development and progression, and leukocytes contribute to the development of many solid tumors, including breast cancer [40]. Genetic studies in mouse models have demonstrated the importance of macrophages and T lymphocytes for breast cancer progression [41,42], and the presence of macrophages in human breast tumors correlates with poor prognosis [43]. Therefore, it is possible that the lymphocyte accumulation observed in the *Perp*-deficient mammary epithelium could result in microenvironmental changes that potentiate mammary epithelial tumorigenesis.

We showed previously that loss of Perp in mice promotes both the initiation and progression of UVB-induced skin cancer [17]. To determine whether Perp contributes to tumor suppression in other epithelial cancers, we investigated the effect of *Perp *deficiency on mammary carcinogenesis. To date, few studies have examined the specific connection between desmosomes and breast carcinogenesis. A couple of exceptions are studies in which the desmosomal protein Dsc3 was found to be downregulated during human breast cancer development [44], and in which Dp expression was observed to be inversely correlated with human breast tumor growth and progression [45]. To more directly assess how desmosomes contribute to carcinogenesis, it is important to evaluate genetic experimental models in which desmosome gene expression can be manipulated in the context of cancer. Here, we found that decreased *Perp *dosage in the mammary epithelium reduces tumor-free survival and tumor latency in *K14-Cre/+;p53^fl/fl ^*mice. Although Perp has roles both in intercellular adhesion and p53-dependent apoptosis, the effects of *Perp *deficiency on tumor latency and tumor-free survival in this mouse cancer model likely result from altered desmosome-mediated cell-cell adhesion, because the tumors are null for *p53*. Collectively, our results therefore indicate that Perp can display tumor-suppressor activities in more than one type of epithelial cancer and that *Perp *deficiency can promote tumor development in the context of different tumor-promoting stimuli. In the future, it would be interesting to investigate how combined targeting of adherens junctions and desmosomes would affect tumorigenesis in this model system.

Our results demonstrate reduced expression of Perp protein in a variety of human breast cancer cell lines, as compared with normal cells, suggesting the possibility that Perp downregulation may contribute to cancer progression. Interestingly, a previous study by Neve *et al. *[46] identified characteristics that led to the segregation of different normal mammary and breast cancer cell lines into functionally distinct subtypes, described as Luminal, Basal A, and Basal B. Some of the cell lines were evaluated for invasive behavior in a Boyden chamber assay, with the most samples being of the Basal B subtype. When we overlap our data describing Perp levels in these same cell lines with the Boyden chamber results of Neve *et al.*, it suggests that Perp levels may inversely correlate with an invasive phenotype. For example, the nontransformed MCF10A cells exhibited high Perp levels but no invasion, whereas the BT549, MDA-MB-231, and SUM149PT breast cancer cells expressed comparatively low Perp levels and displayed significant invasive activity. Together, the two studies suggest that reduced Perp expression may be one characteristic that contributes to the invasive behavior of breast cancer cells and breast cancer progression *in vivo*.

Analyses of human cancers have suggested the importance of Perp as a prognostic marker, as *Perp *downregulation is associated with particularly aggressive uveal melanomas [47] and is predictive for esophageal cancers that will fail to respond efficiently to preoperative combination chemotherapy and radiation treatment [48]. Moreover, Perp loss correlates with increased rates of local relapse in human head and neck squamous cell carcinoma patients (personal communication, Quynh-Thu Le, M.D.). Although patient outcome has not yet been correlated with Perp expression levels in human breast cancer, human *Perp *(also known as *THW*) is downregulated in human mammary carcinoma cell lines compared with nonmalignant mammary epithelium [49]. In addition, the chromosomal region to which human *Perp *maps -- 6q24 -- is deleted in human breast cancer, and loss of heterozygosity at this region has been detected both in breast carcinoma cell lines and in human breast tumors [49,50]. Future studies will better elaborate the mechanisms by which Perp suppresses epithelial cancers and will evaluate Perp as a prognostic indicator or therapeutic target in breast cancer.

## Conclusions

Our findings demonstrate for the first time the expression of the critical desmosome protein, Perp, in the mammary epithelium and suggest an adhesive role for Perp in this context, which may contribute to maintaining homeostasis in this tissue. Furthermore, our results show that *Perp *deficiency promotes mammary tumorigenesis, thereby implicating Perp as a suppressor of mammary cancer in a mouse model. Taken together with our data demonstrating reduced Perp expression in many human breast cancer cell lines relative to untransformed cells, our findings suggest the importance of further investigation of Perp as a marker for breast cancer staging, prognostication, or treatment.

## Abbreviations

Dsg: desmoglein; Dsc: desmocollin; Pg: plakoglobin; Dp: desmoplakin; K8: keratin 8; K14: keratin 14; MEC: mammary epithelial cell; Perp: p53 apoptosis effector related to PMP-22; SMA: smooth muscle actin.

## Competing interests

The authors declare that they have no competing interests.

## Authors' contributions

RLD conceived and designed all of the experiments, performed the majority of the experiments, data analysis, and statistical analysis, and drafted the manuscript. JLB contributed substantially to the experimental design, assisted with experiments and provided technical expertise, performed data analysis, and helped to draft the manuscript. HV and ADB contributed to the data analysis of mammary gland and mammary tumor histology and helped with the design and execution of CD45 immunohistochemistry experiments. SB performed some of the Western blots to define levels of desmosome proteins in wild-type and *Perp-/- *MECs and provided critical feedback on the manuscript. MJB participated in the design of the study and its coordination. LDA conceived of the study and designed experiments, analyzed data, and contributed to writing the manuscript. All authors read and approved the final manuscript.
